# Clinical presentation and outcomes of non-typhoidal *Salmonella* infections in patients with cancer

**DOI:** 10.1186/s12879-021-06710-7

**Published:** 2021-09-29

**Authors:** Nobuyoshi Mori, Ariel D. Szvalb, Javier A. Adachi, Jeffrey J. Tarrand, Victor E. Mulanovich

**Affiliations:** 1 Department of Infectious Diseases, Infection Control and Employee Health, The University of Texas MD Anderson Cancer Center, 1515 Holcombe Boulevard, Unit 1460, Houston, Texas 77030 USA; 2 Department of Laboratory Medicine, The University of Texas MD Anderson Cancer Center, 1515 Holcombe Boulevard, Unit 0084, Houston, Texas 77030 USA; 3 Present Address: Division of Infectious Diseases, St. Luke’s International Hospital, 9-1 Akashi-cho, Chuo-ku, Tokyo, 104-8560 Japan

**Keywords:** *Salmonella* infections, Foodborne diseases, Febrile neutropenia, Immunocompromised host, Sepsis, Bacteremia

## Abstract

**Background:**

Non-typhoidal *Salmonella* (NTS) infection is thought to be more severe in cancer patients, but this has not been studied since the development of new cancer therapies, increasing antibiotic resistance and the introduction of new antibiotics. We sought to describe the demographic characteristics, microbiological findings, clinical manifestations, and outcomes of NTS infections in cancer patients at our institution.

**Methods:**

We reviewed microbiology laboratory records and identified patients who had cancer and from whom NTS organisms were recovered between January 1, 2000 and December 31, 2013, at a comprehensive cancer center in Houston, Texas. Descriptive statistics were used to summarize patient characteristics, clinical presentation and outcomes.

**Results:**

We identified 110 isolates from 82 patients with 88 episodes of NTS infection (including five relapses [6%] in four patients, and two consecutive episodes in one patient). Fifty-five patients (67%) had hematologic malignancies. Most NTS isolates were susceptible to the commonly prescribed antimicrobials. Sixty-nine percent of patients had sepsis and one-third had severe sepsis or septic shock. Gastroenteritis, bacteremia, or both were present in 69% of patients, and the rest had focal infection. Mortality at 30 days was low (8%). Relapses occurred only in patients receiving ≤ 10 days of antibiotic therapy.

**Conclusions:**

NTS affects predominantly patients with hematologic malignancies, followed by gastrointestinal and genitourinary cancers. Invasive disease, sepsis, and septic shock are common presentations among admitted patients. Antimicrobial prophylaxis may not prevent NTS infection. Thirty-day mortality and attributable mortality rates were low in our series compared to older case series. Early appropriate antibiotic therapy may have had a role in decreasing mortality. Relapses occurred in patients receiving ≤ 10 days of therapy, suggesting the need for longer duration of antibiotic therapy in cancer patients with uncomplicated NTS infections.

## Background

Non-typhoidal *Salmonella* (NTS) is the leading cause of illness, hospitalizations, and death among foodborne pathogens in the United States [1]. In immunocompetent hosts, NTS infection presents most commonly as diarrheal illness, usually self-limited. Factors that increase the risk for NTS infection include extremes of age, reduced gastric acid secretion due to atrophic gastritis, gastric surgery, or medications (H2 receptor antagonists or proton pump inhibitors), and use of antibiotics in the prior month [2–4]. Immunosuppressed patients are at increased risk of NTS infection, particularly severe disease such as septicemia and disseminated infection [3, 5]. In 1967, Han et al. reported that salmonellosis occurred more frequently in cancer patients than in other patients at his institution [6], and in 1969, Cherubin et al. reported that 41% *Salmonella* septicemias in New York City occurred in patients with cancer [7].

Although NTS infection in cancer patients has been described in Southeast Asia [5, 8, 9], the largest case series were published in Europe in 1994 [10] and in the United States in 1971 [11]. Since then, considerable improvements in cancer therapy and supportive care, as well as new antimicrobials and increasing antibiotic resistance, have altered the landscape in which these infections occur. The aim of the present study was to describe the demographic characteristics, prevalence of risk factors previously reported in the literature, microbiological findings, clinical manifestations, and outcomes of NTS infections in patients with cancer in our institution.

## Methods

### Hospital setting, bacterial isolates, and data collection

The study was conducted at a comprehensive cancer center in Houston, Texas, after approval by the Institutional Review Board. Our microbiology laboratory receives and processes all clinical cultures. We therefore reviewed our laboratory records to identify patients from whom NTS organisms were recovered between January 1, 2000 and December 31, 2013. At that time, we had not yet instituted stool testing with a multiplex PCR panel for enteric pathogens. Thus, NTS from these patients was identified from cultures using Vitek-2 or Vitek MS (bioMérieux, Durham, North Carolina). The serogroups of *Salmonella* isolates were determined by O antisera (Difco Laboratories, Detroit, Michigan) using the slide agglutination test and confirmed by the Houston Health and Human Services Department.

We retrospectively reviewed the medical records of all patients with cancer who had positive cultures for NTS during the period studied. Data extracted from the medical record included demographic characteristics, cancer diagnosis, other underlying diseases, use of immunosuppressant and chemotherapeutic agents, antimicrobial prophylaxis, clinical manifestations, severity of disease, site of infection, NTS serogroup, antimicrobial susceptibilities, treatment, and outcomes.

### Definitions

Infection with NTS was defined as the presence of clinical manifestations, including systemic and/or localized signs or symptoms, along with recovery of the organism from clinically infected sites such as blood, stool, urine, sputum, or other normally sterile sites. An episode of NTS infection included all positive cultures from any site within a 2-week period. In order to differentiate relapse from persistent infection, re-infection with the same NTS serotype, or asymptomatic carrier state, we defined relapse as a new positive culture(s) with the same NTS serotype occurring ≥ 2 weeks and ≤ 3 months from the previous positive cultures in a patient who received therapy with an antibiotic to which the isolate was reported sensitive. Consecutive infections occurred when a patient developed ≥ 2 episodes of infection with different serotypes of NTS, regardless of the time between them.

Episodes of NTS infection were classified as (1) acute gastroenteritis (GE), if the patient presented with diarrhea (with or without other gastrointestinal symptoms or fever) and had positive stool cultures but no evidence of extra-intestinal organ involvement or positive blood cultures; or (2) invasive disease, comprising three subcategories based on the classification proposed by Ramos et al. [12]: (a) GE with bacteremia, if the patient presented with diarrhea (with or without positive stool cultures) within a month of positive blood cultures but without focal infection; (b) primary bacteremia, when NTS was isolated from blood cultures in a patient without GE or evidence of focal infection; and (c) focal infection, when an organ or system was affected based on clinical and/or radiographic findings along with positive cultures from the affected organ (e.g., lungs, urinary tract, biliary tract, musculoskeletal system) with or without GE or bacteremia.

Use of gastric acid-reducing agents included administration of H2-blockers or proton pump inhibitors within 30 days of infection. Immunosuppressive therapy included use of corticosteroids (high dose was defined as ≥ 600 mg of prednisolone or its equivalent, per month), cytotoxic chemotherapy, a calcineurin inhibitor, or TNF-alpha blockade within 30 days of infection.

Fever was defined as body temperature > 38 °C. Neutropenia was defined as absolute neutrophil count ≤ 500 cells/µL and lymphopenia as an absolute lymphocyte count ≤ 500 cells/µL. Splenomegaly was defined based on radiographic findings as spleen measurement > 11 cm.

Sepsis was defined as systemic inflammatory response syndrome caused by infection with ≥ 2 of the following findings: pulse rate > 90 beats/minute, respiratory rate > 20 breaths/minute, body temperature > 38 °C or < 36 °C, or white blood cell count of > 12,000/µL or < 4000/µL. Severe sepsis was defined as sepsis with hypotension responsive to fluid boluses. Septic shock was defined as hypotension not responsive to fluid boluses, requiring use of vasopressors and/or accompanied by organ failure [13].

## Results

We identified from the microbiological records 112 isolates of NTS collected between January 1, 2000, and December 31, 2013, corresponding to 84 individual patients. Two patients with one isolate each did not have a diagnosis of cancer and were excluded. The remaining 110 isolates corresponded to 82 cancer patients with 88 episodes of NTS infection, including five relapses (6%) in four patients and a second consecutive episode of infection in one patient (the first infection with serotype C1 and the second with serotype F).

### Patient demographics

Demographic details are shown in Table 1.

**Table 1:** Demographic and clinical characteristics of patients with non-typhoidal *Salmonella* infection (N = 82)

Characteristic	N (%)
Median age (range)	55 years (1–83 years)
Female sex	38 (46)
Hematologic malignancy	55 (67)
Acute leukemia/Myelodysplastic syndrome	18
Chronic leukemia	8
Multiple myeloma	9
Lymphoma	20
Solid tumors	27 (33)
Gastrointestinal	7
Genitourinary	5
Breast	4
Lung	4
Head and neck	3
Nervous system	3
Melanoma	1
Hematopoietic stem cell transplantation	14 (17)
Autologous	7
Allogeneic	7

### Microbiological findings

Among the 110 isolates, 51 (46%) were obtained from blood cultures, 27 (25%) from stool, 19 (17%) from urine, and 13 (12%) from other sites. The most common serogroup isolated was C, with 38 isolates (35 %), followed by B with 28 isolates (25%), and D with 26 isolates (24%). Serogroups E, F, G, and H comprised the remaining 18 isolates (16%). Almost all isolates were susceptible to the commonly prescribed antimicrobials against this infection. Eleven of 98 isolates tested (11%) were resistant to ampicillin, 3 of 101 (3%) to ciprofloxacin, and 4 of 100 (4%) to trimethoprim/sulfamethoxazole. All isolates tested were susceptible to ceftriaxone, cefepime, and imipenem.

### Clinical characteristics

The clinical characteristics of 88 episodes of NTS infection are presented in Table 2. Patient factors that impair host defenses included lymphopenia and neutropenia, present within 30 days prior to infection in 28% and 13% of episodes, respectively, and during infection in 52% and 25%, respectively. Fifty-six percent of patients took medications that reduced gastric acid secretion, a barrier to *Salmonella* infection. Medications that impaired the immune response, such as corticosteroids (49%) or active chemotherapy and other immunosuppressants (66%), were also common within 30 days of an infection episode. Patients had received antibiotics within 30 days prior to a positive culture in 40% of episodes, including long term antibiotic prophylaxis at the time of diagnosis in 11 episodes (13%), of which four each received fluoroquinolones and trimethoprim/sulfamethoxazole and three cefpodoxime. We could not verify compliance with prophylaxis from the chart. Only one of the 11 isolates was resistant to the prophylactic antibiotic being prescribed (trimethoprim/sulfamethoxazole).

**Table 2:** Clinical characteristics of 88 episodes of non-typhoidal *Salmonella* infection

Clinical characteristics	N (%)
Host Factors	
Neutropenia in previous 30 days	11 (13)
Neutropenia during infection	22 (25)
Lymphopenia in previous 30 days	25 (28)
Lymphopenia during infection	46 (52)
Medications within 30 days of infection	
Gastric acid suppression	49 (56)
Corticosteroids	43 (49)
Active chemotherapy	58 (66)
Antibiotics	35 (40)
Clinical presentation	
Fever	51 (58)
Diarrhea	48 (55)
Nausea/vomiting	40 (45)
Abdominal pain	37 (42)
Urinary symptoms	9 (10)
Sepsis syndrome	
Sepsis	32 (36)
Severe sepsis	24 (27)
Septic shock	5 (6)
Outcomes	
Hospital admission	64 (73)
Intensive Care Unit admission	11 (13)
Mechanical ventilation	3 (3)
All-cause mortality at day 30	7 (8)

The most common symptoms at presentation included fever (58%), diarrhea (55%), nausea/vomiting (45%), abdominal pain (42%), and urinary symptoms, such as dysuria, urinary frequency, or lower back pain (10%). Most patients had sepsis syndrome (69%), with 33% presenting with severe sepsis or septic shock, occurring with all clinical presentations of NTS (Table 3).

**Table 3:** Occurrence of severe sepsis and deaths by clinical presentation

Clinical presentation	No. (%)		
	Episodes	Severe sepsis or septic shock	Death within 30 days
Acute GE	22 (25)	8 (36)	0 (0)
Acute GE with bacteremia	12 (13)	6 (50)	1 (8)
Primary bacteremia	27 (31)	10 (37)	2 (7)
Focal infection	27 (31)	5 (19)	4 (15)
Total	88 (100)	29 (33)	7 (8)

GE, Gastroenteritis

Table 3 shows the episodes of infection classified according to previously defined clinical syndromes. Twelve of the 34 episodes (35%) with acute GE had bacteremia, whereas 22 of 88 episodes (25%) had disease limited to the gastrointestinal tract. There were 27 episodes of primary bacteremia and 27 of focal infection. The most serious episodes of focal infection included five cases of musculoskeletal infection, four of them with bacteremia but none with acute GE; two cases of pneumonia, one of them with acute GE; and one case of bone marrow infection that relapsed and required prolonged intravenous therapy.

Urinary tract infection (UTI) was the most common focal infection: sixteen patients had 18 episodes, including one relapse and one with consecutive infections with different strains of NTS. Six of these patients (38%) had one or more urinary tract abnormalities or a foreign body, including nephrolithiasis (4), hydronephrosis (3), and one each with history of recurrent UTIs, urinary incontinence, vesicoureteral reflux, ureteral stent, and placement of an Indiana pouch. Only two of them had acute GE and none had bacteremia.

### Management and outcomes

Most episodes required hospitalization (73%) and 13  required admittance to the intensive care unit, although only 3% required mechanical ventilation. Antimicrobial therapy was documented in 79 episodes (90%), with a median duration of 14 days (range 1–61 days). Antibiotics were initiated the same day cultures were obtained in 64 of 79 (81%) episodes. Initial therapy included cephalosporins in 34 episodes, fluoroquinolones in 24, carbapenems in 11, beta lactam/beta lactamase inhibitors in 9, and trimethoprim/sulfamethoxazole in 1. Appropriate antimicrobial therapy was started within 24 h of cultures being obtained in 35 of 43 (81%) episodes of bacteremia and within 72 h in 40 of 43 (93%) episodes.

Among nine episodes (10%) with no antimicrobial therapy documented, one patient died of progressive brain cancer with intracranial hemorrhage the day the urine culture was obtained. One patient had bacteremia but was not hospitalized and the infection was regarded as a contaminant by the primary oncologist. Three patients had acute GE and five had UTI. It is not clear if these patients received therapy outside our institution. Surgical intervention with percutaneous drainage was performed in all four episodes with musculoskeletal abscess formation.

One patient with acute GE had two relapses, and one patient each with UTI, acute GE, and bone marrow infection had one relapse. Patients who had a relapse received a shorter course of antibiotic therapy for the initial infection with a median duration of 7 days (range 2–10 days), compared with a median of 14 days for all treated patients. Five of 20 episodes treated for ≤ 10 days (25%) resulted in a relapse compared with none in those treated for longer than 10 days. One patient had two episodes of UTI with different NTS serotypes (reinfection).

Seven patients died within 30 days of infection, all had invasive NTS disease, but four died of cancer progression and NTS was considered a contributing factor to death in the remaining three cases. Among these three patients, one died of disseminated fungal infection proven by autopsy, one of graft-versus-host disease causing hepato-renal failure, and one of polymicrobial bacteremia and sepsis that included NTS. NTS was considered one of the principal contributors to death only in the last case.

## Discussion

Our data indicated that NTS continues to cause severe infections in cancer patients, particularly in those with hematologic malignancies [14]. Patients with cancer have an increased risk of acquiring NTS infection because of mucosal barrier disruption due to cancer or its therapy [15], immunodeficiency due to cancer itself and receipt of cytotoxic chemotherapy and/or corticosteroids [3, 14, 16]. Other previously described factors such as gastric acid suppression, use of antibiotics in the prior month, and old age were also highly prevalent among our patients [2, 4, 8]. Hematologic malignancies account for 10% of the annual incidence of cancer in the United States [17], however, they constituted two-thirds of our patients with NTS infection and 48% and 60% in previous case series [10, 11]. Gradel et al. reported that blood cancers might increase the risk of *Salmonella* infection by a factor of 5–10 when comparing patients in the Danish Cancer Registry with matched controls, whereas patients with solid tumors had an increased risk of only 1–1.5 times that of controls [18]. Blood cancers are associated with prolonged and profound cellular and humoral immunodeficiency. Improved survival and stem cell transplantation leading to long-term immunosuppression may increase chances of exposure and susceptibility to infection.

Among patients with solid tumors, upper or lower gastrointestinal malignancies were most common (26%). These patients may have mucosal barrier disruption, lack of gastric acidity, and other anatomic or physiologic abnormalities that facilitate infection through this most common portal of entry. Genitourinary malignancies were second most common (19%). This is not surprising, because structural abnormalities of the urinary tract caused by the cancer or its treatment, as well as nephrolithiasis, obstruction, and renal transplantation, have been previously described in most patients with NTS UTI [19], and these factors likely favor NTS infection of the urinary tract.

As expected, there was a wide distribution of NTS serogroups, given that patients most likely acquire the infection sporadically in the community. Our isolates showed low to moderate levels of antimicrobial resistance, in contrast to reports of increasing resistance in the United States [20]. Drug resistance has been associated with excess bloodstream infections and hospitalizations [21], including resistance rates to ceftriaxone of 2.9% between 1996 and 2013 [22].

One surprising finding was the occurrence of NTS infection in patients receiving long-term prophylactic antibiotics to which the organism was sensitive in 10 cases, however, we could not verify the total duration or compliance with these antibiotics. Possible reasons include noncompliance with antibiotic prophylaxis, breakthrough infection due to high inoculum, or severe immunosuppression, as well as partial effectiveness of the prophylaxis.

Most of our patients presented with sepsis, and one-third had severe sepsis or septic shock, with 13% requiring admission to the intensive care unit. GE, bacteremia, or both were present in 69% of patients, and the rest had focal infection, most commonly UTI. We found that four episodes included musculoskeletal infection that required abscess drainage.

The mortality rate in our series was lower than expected based on prior reports and severity of illness, with an overall 30-day mortality rate of 8%. NTS infection contributed in three of the 7 patients who died (4%) and was considered a significant contributor in one patient. Noriega et al. reported a higher 30-day mortality rate (23%) and attributable mortality rate (15%) among 40 patients with *Salmonella* infections in a cancer center in Belgium from 1975 to 1990 [10].

NTS bacteremia has high mortality rates even in modern times. Three of 43 bacteremic patients (27 with primary bacteremia, 12 with acute GE with bacteremia and 4 with musculoskeletal infection and bacteremia) in our series (7%) died within 30 days. A study from Taiwan reported an in-hospital mortality rate for NTS bacteremia of 41% in patients with cancer and 18% in those without [8], and a Malaysian study reported an overall mortality rate of 31% in patients with cancer compared with 5% in others [5]. Owing to different definitions, these numbers are not directly comparable but serve as a reference. Patients with advanced cancer or its complications may have high mortality rates independent of NTS infection, as seen in our study.

Delay in initiating appropriate antibiotic therapy in patients with sepsis and septic shock is associated with increased mortality [23]. Yen el al reported that inadequate antibiotic treatment of NTS bacteremia was an independent risk factor predicting mortality by multivariate logistic regression analysis [9]. Only 71 % and 62% of patients with NTS bacteremia in two studies received appropriate antimicrobials within 72 h [5, 8], compared to 81% (35 of 43) of our bacteremic patients receiving appropriate antimicrobials within 24 h and 93% (40 of 43) within 72 h. Empiric antimicrobial therapy for cancer patients with sepsis admitted to our institution includes third- or fourth-generation cephalosporins or carbapenems (no NTS resistance found in our study). The use of early and appropriate empiric antibiotic therapy may have contributed to improved outcomes.

The recommended duration of therapy for NTS GE in immunosuppressed individual is 3–7 days, 10–14 days for NTS bacteremia, and longer duration (4–6 weeks) is recommended for complicated infections such as musculoskeletal (plus surgical drainage, if indicated), endarteritis, or endocarditis [16]. One quarter (5 of 20) of our patients who received antibiotic therapy for ≤ 10 days relapsed, compared with none of the 68 treated for more than 10 days. Our cohort was not large enough to allow statistical comparisons; however, these findings suggest that a longer course of therapy for NTS may be needed to prevent relapse in patients with cancer. Noriega et al. reported that five of six patients with *Salmonella* infections in a cancer center in Belgium who experienced relapse received less than 10 days of therapy [10].

Our study had several limitations. It was based in a comprehensive cancer center were the severity of illness and predisposing factors such as degree of immunosuppression may be higher than in patients with cancer in the community. Secondly, its retrospective nature and the number of patients relative to the number of variables to consider did not allow for certain statistical comparisons that may have given more strength to our conclusions.

## Conclusions

NTS is a cause of severe infections in cancer patients, particularly in those with hematologic malignancies, followed by patients with gastrointestinal and genitourinary cancers. Invasive disease, sepsis, and septic shock are common among admitted patients. Antimicrobial prophylaxis may not prevent NTS infection. Thirty-day mortality and attributable mortality rates were low in our series compared to older case series. Early appropriate antibiotic therapy may have had a role in decreasing mortality. Relapses occurred in patients receiving ≤ 10 days of therapy, suggesting the need for longer duration of antibiotic therapy in patients with cancer and uncomplicated NTS infections.

## Data Availability

The datasets generated and/or analyzed during the current study are not publicly available due to patient confidentiality but are available from the corresponding author on reasonable request.

## Abbreviations

NTS: Non-typhoidal *Salmonella*
GE: Acute gastroenteritis
TNF: Tumor necrosis factor
UTI: Urinary tract infection
